# Current role of systematic biopsy in diagnosis of clinically significant prostate cancer in primary combined MRI-targeted biopsy: a high-volume single-center study

**DOI:** 10.1007/s00345-022-04230-w

**Published:** 2022-12-07

**Authors:** Philipp Krausewitz, Dorothea Fostitsch, Richard Weiten, Niklas Kluemper, Johannes Stein, Julian Luetkens, Glen Kristiansen, Jörg Ellinger, Manuel Ritter

**Affiliations:** 1 Department of Urology and Pediatric Urology, University Medical Center Bonn (UKB), University Hospital Bonn, Bonn, Germany; 2 Institute of Experimental Oncology, University Medical Center Bonn (UKB), Bonn, Germany; 3 Department of Diagnostic and Interventional Radiology, University Medical Center Bonn (UKB), Bonn, Germany; 4 Institute of Pathology, University Medical Center Bonn (UKB), Bonn, Germany

**Keywords:** Clinically significant prostate cancer, Biopsy method, Perifocal saturation biopsy; adjacent sectors, Systematic biopsy

## Abstract

**Purpose:**

Additive systematic biopsy (SB) contributes to prostate cancer (PCA) detection in MRI-targeted biopsy (TB). However, the reasons for this are not yet clear. We compared the performance of TB, SB and the combined approach (CB) in biopsy-naive men to determine the added value of SB for tumor grading and spatial tumor distribution.

**Methods:**

Two hundred and fifty-nine men with PI-RADS 3–5 graded lesions who underwent CB were enrolled. Data were prospectively collected, and cancer detection rates (CDR) were compared at patient and lesion level. Gleason grade up- and down-grading from biopsy to prostatectomy specimens (*n* = 56; 21.6%) were determined. Clinically significant cancer (csPCA) was defined as Gleason grade ≥ 2.

**Results:**

CDR by CB based on PI-RADS categories 3, 4 and 5 for PCA were 24%, 72% and 98% and 17%, 64% and 96% for csPCA. CB detected more PCA and csPCA than TB (*p* < 0.001). However, TB showed higher efficiency, defined as CDR per biopsy core, for PCA and csPCA in PI-RADS 4–5 rated patients (*p* < 0.001). Concordance between biopsy and prostatectomy grading was highest in CB with misdiagnosis of csPCA in 25% of men. TB missed cancer attributed to the index lesion in 10.2% and underestimated csPCA in 7%. In these cases, 76% of csPCA were detected and 85% were upgraded to csPCA by SB in adjacent sectors.

**Conclusion:**

SB cannot be safely abundant without increased diagnostic uncertainty. When TB missed csPCA, SB detected it close to the MRI-target lesion. Therefore, perifocal biopsies could potentially replace 12-core SB with increased efficiency in taking manageable risks.

**Supplementary Information:**

The online version contains supplementary material available at 10.1007/s00345-022-04230-w.

## Introduction

In accordance with prostate cancer (PCA) lethality, discrimination between clinically nonsignificant PCA (nsPCA; defined as ISUP grade group 1) and clinically significant PCA (csPCA; defined as ISUP grade group ≥ 2) is an important goal. Inter- and intratumoral heterogeneity, however, complicates risk stratification during diagnostic workup. High-quality studies in recent years have impressively demonstrated the potential of the multiparametric magnetic resonance (MRI)-pathway using the Prostate Imaging Reporting and Data System (PI-RADS) in PCA evaluation. Increased detection of csPCA, reduction of nsPCA and beneficially reduced number of biopsies compared to systematic biopsy were shown [1–3]. Nevertheless, the MRI-only pathway misses a significant amount of csPCA [4]. Hence, guidelines recommend the combined approach (CB) of MRI-guided biopsy (TB) and systematic biopsy (SB) during the initial investigation [5]. Despite advances, the contributing role of SB to TB remains a matter of debate. [1, 4, 6]. Advocates of CB proclaim an increased value for the true tumor grading and extent, which leads to a decreased likelihood of misdiagnosis and enables tailored disease management [7–10]. Still, uncertainty concerning tumor grading after CB needs to be expected in one-third of men [7]. A specific explanation for the improved performance by additional SB is not yet clear. SB might compensate for targeting errors, MRI invisible cancer and under-sampling of the target approach considering the multifocality of PCA lesions [6, 11].

We conducted this retrospective analysis to clarify the distribution and the diagnostic yields of additive SB for the detection of csPCA during the initial prostate biopsy. Our analysis aimed to investigate the performance of SB, TB and CB and the incremental value of each biopsy method for tumor grading and distribution. Moreover, we investigated the efficiency and the impact of PCA surrogate markers (prostate-specific antigen (PSA), PSA-density (PSAD), prostate volume, abnormal digital-rectal examination (DRE) and abnormal transrectal ultrasound findings (US)) on cancer detection rate (CDR). Finally, we determined spatial tumor distribution according to the MRI index lesion.

## Patients and methods

### Patients

Biopsy-naïve men who underwent CB were assembled from a prospectively collected institutional database (University Medical Center Bonn (UKB)) and included in the ethically approved (158/22) retrospective audit. Indications for performing MRI were suspicious PSA, abnormal DRE or abnormal transrectal US. *N* = 259 men with PI-RADS lesions ≥ 3 who underwent MRI-targeted and systematic biopsy were included. Uro-radiologists rated and reported the MRI results according to PI-RADS version 2.1. [12]. The majority of MRI were performed in the UKB (70.7%). More detailed patient characteristics are provided in Supplementary Tables S1 and S2.

### Methods

The same physician performed SB and TB in one session; biopsies were performed by two experienced urologists performing each > 250 prostate biopsies annually (P.K., J.E.). A pre-defined, software-assisted template was used for the institutional standardized biopsy in all patients (Supplementary Fig. S1). Biopsies were conducted under antibiotic prophylaxis, rectal cleansing and local anesthesia. Software-assisted fusion technique (KOELIS Trinity®) was used. All cores were separately documented, collected and histopathologically evaluated. Histopathology was conducted according to guidelines in a reference uropathology [5]. csPCA was defined as any Gleason 3 + 4 or higher PCA (International Society of Urological Pathology (ISUP) Grade Group ≥ 2). The number of positive cores and ISUP grading for any cancer detected was recorded.

CDR was determined primarily per patient and supplementary per lesion and with respect to the number of cores taken. CDR of SB, TB and CB were compared, stratified by PI-RADS score, prostate volume, DRE, transrectal US findings and PSAD. Lesion-level analysis included the SB results for the MRI index lesion and adjacent sectors. The adjacency was defined as any sector adjacent to the sector with a PI-RADS 3–5 lesion in the transverse or craniocaudal direction. The sectorial division was defined by PI-RADS v2.1 sector map. The subset of patients with divergent SB and TB results in terms of PCA detection, csPCA detection and PCA staging was classified, and tumor distribution was reported. In addition, in a subgroup of our study cohort who had undergone radical prostatectomy at UKB (*n* = 56), we compared the histopathologic results of the prostatectomy specimen with the previous biopsy result.

### Statistics

All data were coded and analyzed with “IBM SPSS Statistics,” v26. Descriptive statistics included frequencies and proportions for categorical variables. Medians and interquartile ranges (IQRs) were reported for continuously coded variables. Differences were detected using the *T* test for independent samples, chi-square tests or McNemar paired test. *p* values of ≤ 0.05 were considered statistically significant.

## Results

PCA was detected in 71.8% with a prevalence of csPCA in 65.6% and nsPCA in 6.2% of all cases. In total, 993 of 3546 (28.0%) biopsy cores were tumor-bearing, distributed between SB (*n* = 755/3108; 24.3%) and TB (*n* = 238/438; 54.3%). After the procedure, 0.8% of patients suffered a urinary tract infection. More detailed patients’ characteristics are provided in Supplementary Tables 1, 2 and 3.

**Fig. 1:**
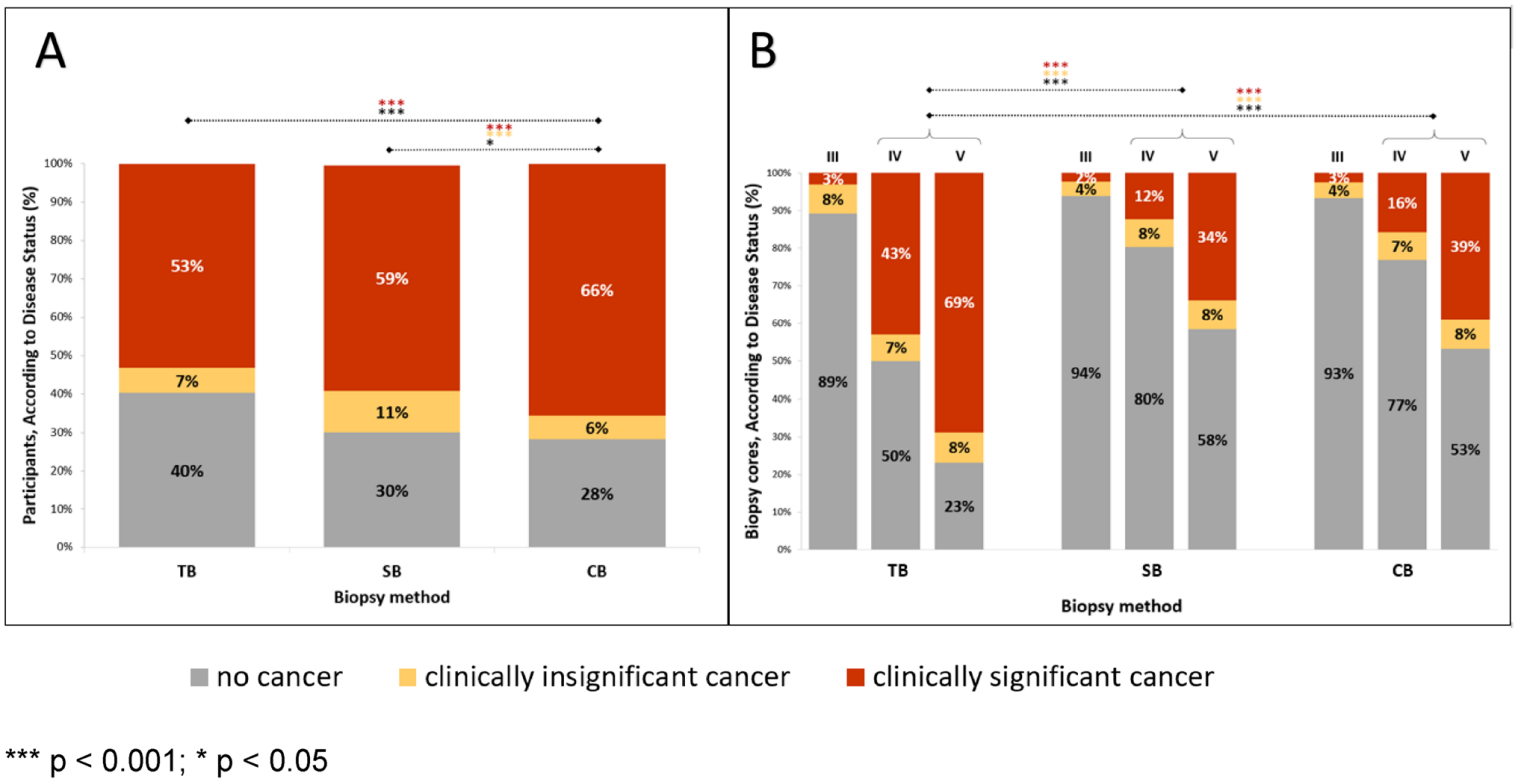
Cancer detection and diagnostic efficiency of systematic biopsy, MRI target biopsy and the combined approach. ***p < 0.001; *p < 0.05. Figure 1 shows cancer detection rates according to biopsy method (**A**) and cancer detection rates per biopsy core taken (**B**) by the MRI targeted biopsy (TB), systematic 12-core biopsy (SB) and the combined approach (CB). Categorical data are presented as %.

Highest PI-RADS 3–5-dependent CDR were determined for CB: in detail, 23.8%, 71.5% and 97.5% for PCA and 16.7%, 64.2% and 96.2% for csPCA. Overall CDR for PCA and csPCA by CB were significantly higher than those of TB (*p* < 0.001 for both) and SB (*p* = 0.016 and *p* < 0.001, respectively), Fig. 1.

### Impact of PCA surrogates

PCA and csPCA detection by CB, TB and SB were significantly increased in accordance with abnormal DRE and suspicious transrectal US findings (both *p* < 0.001 for all). Moreover, a predictive value for PCA and csPCA detection by TB and CB was determined for elevated PSAD > 0,1 ng/ml^2^ and > 0,15 ng/ml^2^ and decreased prostate volume < 50 ml, all *p* < 0.001. nsPCA detected by SB was significantly more frequent in men in the absence of suspicious palpation and PSAD < 0.15 ng/ml^2^ (*p* = 0.005 and 0.01, respectively), Supplementary Fig. S2.

### MRI-pathway vs. SB

TB alone led to cancer diagnosis in 83.3% (155 of 186) of patients and detected less nsPCA (6.8%) than SB (10.8%); however, differences were not statistically significant (*p* = 0.065). The absolute PIRADS 3–5-dependent CDR was 16.7%, 56.2% and 88.7% for PCA and 4.8%, 49.6% and 85.1% for csPCA. Comparing PI-RADS 3–5-dependent efficiency, defined as CDR per biopsy core taken, between biopsy methods, TB showed significantly higher efficiency for PCA and csPCA detection than SB or CB, Fig. 1.

Overall CDR for PCA and csPCA by SB was 69.5% and 58.7%, 23.8% and 16.7% for PI-RADS 3, 68.6% and 54.7% for PI-RADS 4 and 95.0% and 81.4% for PI-RADS 5 assessment categories. SB alone detected 180 of 186 (96.8%) of all PCA-diagnosed patients. Despite the absolute increase in cancer detection, no significant differences depending on PI-RADS grades were found for csPCA between SB and TB.

### Concordance of intermethod and postoperative tumor grading

TB and SB simultaneously detected PCA in 148/186 (79.5%) patients, but tumor grading by TB and SB matched only in 90/148 (63%) men. PCA, csPCA and nsPCA were solely diagnosed by SB in 32 of 186 (17.2%), 22/170 (12.9%) and 10/16 (62.5%) patients. SB upgraded nsPCA to csPCA in 11/148 (7.4%) or high-risk PCA, defined as ISUP ≥ 4, in 7/148 (4.7%) men. The incremental value of TB for PCA and csPCA detection was 3.2% (6/186) and 2.9% (5/170), respectively. TB diagnosed only one additional man with nsPCA and upgraded nsPCA in 13/148 (8.8%) and high-risk PCA in 5/148 (3.4%) cases.

Postoperative diagnosis in patients who underwent prostatectomy (*n* = 56) matched with previous results of CB, SB, and TB in 68%, 57% and 43%, respectively. TB, SB and CB underestimated tumor grading at 50%, 36% and 30%. Misdiagnosis of csPCA when comparing biopsy and prostatectomy results was significantly lower in CB (25%) than in TB (54%) and SB (60%).

### Per lesion analysis and spatial distribution of tumor

Significant differences for CDR by TB were shown dependent on index lesion location. PCA and csPCA were more frequently diagnosed in PZ than in TZ (*p* < 0.001 for both). No differences concerning CDR were determined between the predefined locations of SB sampling (Supplementary Figs. S3 and S4). In a PI-RADS-dependent analysis, SB cores however revealed a significantly increased CDR for PCA and csPCA comparing men rated PI-RADS 3 (mean 10.8% ± 1.1 and 4.2% ± 2.4), 4 (mean 19.7% ± 3.7 and 11.7% ± 2.8), and 5 (mean 41.7% ± 6.8 and 33.5% ± 6.8), respectively.

### Tumor distribution concerning MRI index lesion

Where no cancer was attributed to the index lesion by TB (43/418, 10.2%), 30/43 (69.8%) csPCA including 4 high-risk PCA were solely detected by SB. In 1/43 (2.3%) suspicious TB negative lesions, SB revealed cancer (ISUP grade 2) within the target area. 23/42 (54.8%) PCA, 22/29 (75.9%) csPCA and 2/4 (50.0%) high-risk PCA were discovered in adjacent sectors of the MRI lesion, respectively. Non-target, non-adjacent cancer was found in 14 cases including 6 csPCA and one high-risk PCA.

**Fig. 2:**
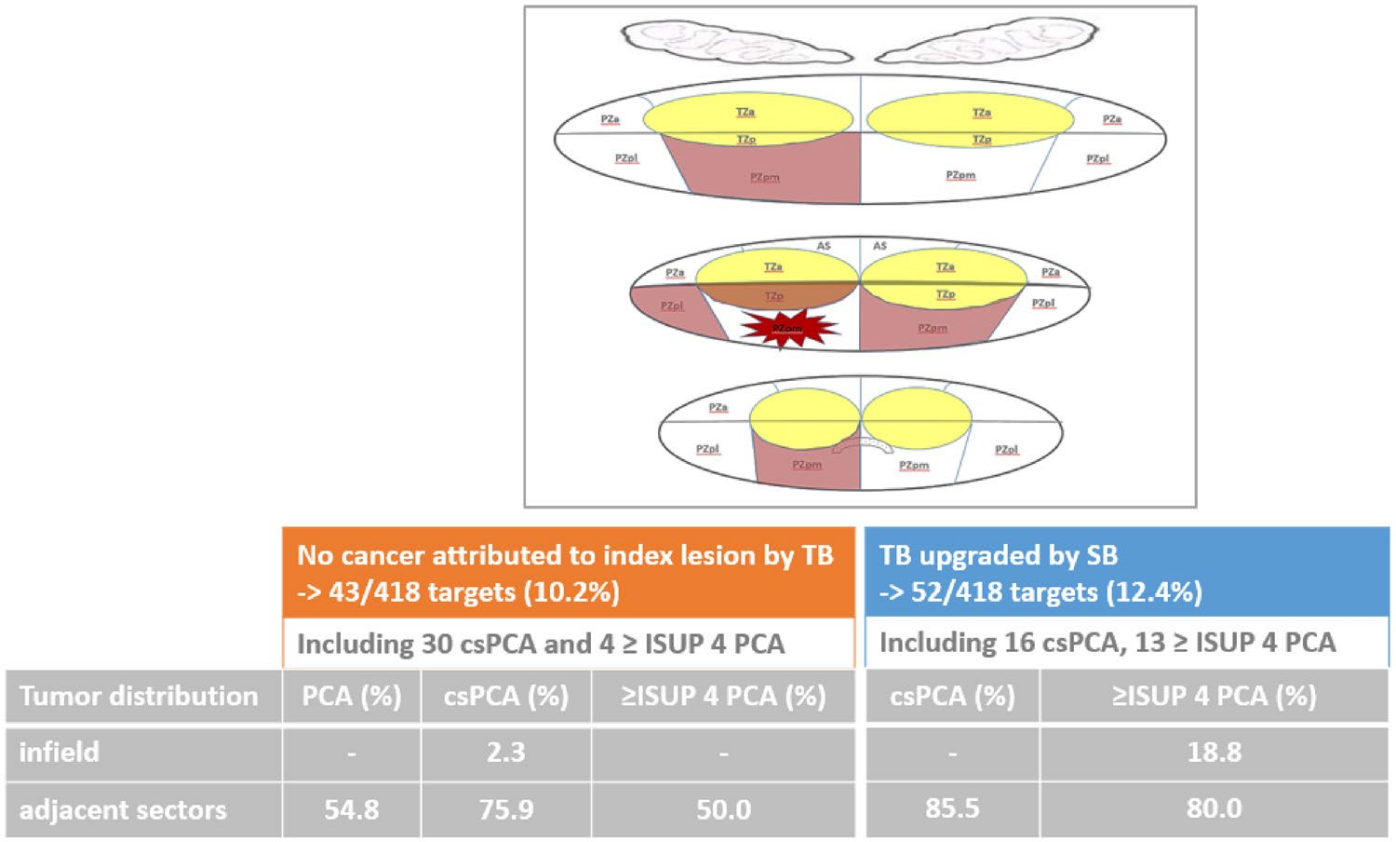
Cancer detection by systematic biopsy and target biopsy according to MRI index lesion. Figure 2 shows the graphical abstract summarizing the analysis at lesion level of the performance of systematic biopsy (SB) and magnetic resonance imaging (MRI) target biopsy (TB) regarding prostate cancer detection (CDR). Tumor grading and spatial tumor distribution for each biopsy method with regards to the MRI index lesion according to PIRADS v2.1 sector map [12] are presented. The adjacency was defined as any sector adjacent to the sector with a PI-RADS 3–5 lesion in the transverse or craniocaudal direction

52/418 TB-detected PCA were upgraded by SB. These 11/52 (21.1%) were upgraded by SB cores on target including three ISUP ≥ 4 tumors. When upgrading was disclosed by SB offside the MRI lesion, 35/41 (85.4%) of the more aggressive PCA were found in adjacent areas and 5/41 (12.2%) were discovered on the contralateral prostate lobe. Hence, TB only approach missed an upgrading to high-risk PCA in 10/41 (24.4%) of the cases, wherein 8/10 (80.0%) were attributed to adjacent sectors and 2/10 (20.0%) to the other prostate lobe, Fig. 2.

## Discussion

Before TB can fully replace SB in biopsy-naïve men for reliable detection and exclusion of csPCA, it is important to define the rationale for optimized CDR by SB during the combined approach.

In line with previous findings, we showed significantly increased detection rates for PCA and csPCA using CB [7–10, 13]. Overall CDR was high (72%) but in the range of PRECISION (71%) [2]. Compared to other series, csPCA detection in our cohort was markedly increased for all biopsy techniques (CB 66%, TB 53% and SB 59%) and nsPCA detection was at the lower end [1–3, 9]. Inclusion of older men (medium age 68.5) with increased tumor burden (abnormal DRE 43%; PI-RADS 5 lesions: 31%, PSA 10.8 ng/ml) and a selection bias by including only MRI-positive men might best explain this. However, PI-RADS 3–5-dependent TB detection rates (16.7%, 56.2% and 88.7% for PCA and 4.8%, 49.6% and 85.1% for csPCA) were in the range of MRI-FIRST and PRECISION. The exceptional net increase of 12.9% csPCA by SB alone in the present study is most likely caused by the standardized software-assisted template SB, as compared to the freehanded random SB, which is error-prone to intra- and interoperator uncertainties [14]. However, additional SB also increased nsPCA detection and potentially induced overtreatment, even if this was not statistically significant.

On the contrary, the underestimation of true tumor grading was high for each solitary biopsy method. Our results corroborate prior prospective series showing the highest concordance with pathological tumor grading for CB (68 vs. 64%) and increasingly diagnostic uncertainties for SB (43 vs. 53%) and TB (57 vs. 47%) [7]. Moreover, a proportional increase of PCA and csPCA detection with clinical and biochemical surrogate markers and inversely proportional relation to prostate volume was confirmed for all biopsy methods [15, 16].

Advantages of MRI-pathway were also evident in our work: omitting SB could have beneficially reduced the number of nsPCA-diagnosed men by 10/259 and unnecessary core sampling could have been safely avoided by nearly two-thirds. Moreover, TB alone showed a significantly raised efficiency for PCA and csPCA detection in overall and PI-RADS-dependent comparison to SB and CB (*p* < 0.001 for all). These findings imply a high potential to reduce biopsy-related distress, anxiety, pain and infectious risk using the TB-only approach [17]. This was at the cost of misdiagnosis of 7.4% csPCA and 4.7% ≥ ISUP 4-graded cancers. Hence, the underestimation of csPCA in our present work was slightly higher than reported in the literature (4.9–5.8% [4, 7, 9]. In line with the systematically reviewed results of Drost et al. TB alone in MRI-positive men missed the diagnosis in 17.2% of men with ISUP grade 2 or higher PCA [4]. CDR of the individual SB cores indicates that a pure reduction of the core number will not improve matters. Rather, it requires the considered adaptation of the biopsy scheme.

Our per lesion analysis confirmed previous findings with an increased CDR for PCA and csPCA in the peripheral zone than in the anterior or transitional zone [18]. When the cancer was upgraded or solely detected by SB, it was utmost attributed to adjacent sectors of MRI index lesion, making an argument for focal perilesional saturation biopsies (FSB) [19, 20]. Performance of TB concerning csPCA detection inside targeted sectors was pleasing with only one missed csPCA and 3 underestimated high-risk cancers.

Despite the low efficiency, the added value of SB for the detection of csPCA was particularly evident in the subgroup of PI-RADS 3 patients, in whom the efficiency of TB was the lowest. Moreover, the benefit of performing additional SB seems to increase inversely to the PI-RADS grading. Consistent with previous findings, our results demonstrate that clinical and biochemical PCA surrogate markers are important factors in deciding whether or not to biopsy a PI-RADS 3 lesion [15, 23].

The TB-only approach including an additional FSB of the adjacent PI-RADS sector map-defined areas would have detected 75.9% (22/29) csPCA and 50% (2/4) high-risk PCA currently determined by additional 12-core SB. Previously series determined that non-suspicious areas adjacent to the MRI index lesion harbor PCA in 69% and csPCA in 52% of all cases, which is almost similar to our results [20, 21]. The present hypothetical TB + FBS approach would have reduced misdiagnosis of TB alone for csPCA and high-risk PCA in 12/13 (92.3%) and 8/10 (80%) cases, respectively. Replacing SB with hypothetical FSB would have been at the cost of missing and/or underestimation of 8/170 (4.7%) csPCA and 4/47 (8.5%) high-risk PCA in the present cohort. On the other hand, saturating the surrounding PI-RADS sectors of the index lesion with one biopsy core each would have resulted in a net reduction of 4–5 cores per patient in the present study (13.7–9.1; − 33.6%). Of note, 92% (11/12) of the misdiagnosed csPCA overseen by FSB were discovered on the contralateral prostate lobe. These results corroborate with recent series exploring the diagnostic yields of FSB [22, 23].

Despite the low efficiency of SB including many unnecessary biopsies, it cannot be eliminated without an increased uncertainty in diagnosis and risk assessment. However, a meta-analysis by Hagens et al. showed a non-inferiority for PCA diagnosis by the TB + regional biopsy approach [20]. Hence, it is conceivable to replace SB with FSB. Furthermore, the FSB results could be even optimized by a perineal approach, as the peripheral zone would be more saturated per biopsy core. The extent and technique of the perifocal biopsy and the necessary amount of on- and peri-lesional biopsies are currently under debate [23–26] and further prospective series are needed to define the optimal spatial biopsy method.

In addition to its retrospective design, limitations of our study include the lack of information on ultimate lesion volume and location on whole mount pathology for the entire cohort. Moreover, no instrumental but a cognitive template was used for standardized institutional 12-core biopsy in all men, which may have led to uncertainty of the sectorial measurement.

Nevertheless, our findings demonstrate the promising detection rate of a possible future alternative biopsy method including regional biopsies of MRI-index lesion adjacent PI-RADS map-defined sectors with increased efficiency for biopsy naïve men at a simultaneously reasonable degree of uncertainty regarding csPCA misdiagnosis. The present results hereby emphasize the importance of metric (prostate volume), clinical (DRE/US) and biochemical (PSAD) surrogate markers of PCA in addition to the PI-RADS category to determine the biopsy method of choice on an individual basis.

## Conclusion

At present, SB cannot be safely abundant without an increase in diagnostic uncertainty. When csPCA is missed by TB, SB detected cancer close to the MRI-target lesion, which suggests that a perifocal saturation biopsy could potentially replace SB with increased efficiency in taking manageable risks.

## Supplementary Information

Below is the link to the electronic supplementary material.

Supplementary file1 (DOCX 413 KB)

## Data Availability

The datasets generated during and/or analysed during the current study are available from the corresponding author on reasonable request.
