# Supplementary material for: Current role of systematic biopsy in diagnosis of clinically significant prostate cancer in primary combined MRI-targeted biopsy: a high-volume single-center study
Source: World J Urol. 2022 Dec 7;41(1):19–25. doi: 10.1007/s00345-022-04230-w (PMC9849165; doi:10.1007/s00345-022-04230-w)
Supplement: Supplementary file 1 — Supplementary file1 (DOCX 413 KB) [file 345_2022_4230_MOESM1_ESM.docx]

**Current role of systematic biopsy in diagnosis of clinically significant prostate cancer in primary combined MRI-targeted biopsy – a high-volume single center study.**

P. Krausewitz^1^, D. Fostitsch^1^, R. Weiten^1^, N. Kluemper^1,2^, J. Stein^1^, J. Luetkens^3^, G. Kristiansen^4^, J. Ellinger^1^, M. Ritter^1^

^1^Department of Urology and Pediatric Urology, University Medical Center Bonn (UKB), Bonn, Germany

^2^Institute of Experimental Oncology, University Medical Center Bonn (UKB), Bonn, Germany

^3^Department of Diagnostic and Interventional Radiology, University Medical Center Bonn (UKB), Bonn, Germany

^4^Institute of Pathology, University Medical Center Bonn (UKB), Bonn, Germany

Address for correspondence: Philipp Krausewitz, M.D.

Department of Urology and Pediatric Urology,

University Hospital Bonn

Email: Philipp.krausewitz@ukbonn.de

Tel.: +4915118853551

Orcid-ID 0000-0002-8213-9975

E-mail address of all authors:

- [Philipp.Krausewitz@ukbonn.de](mailto:Philipp.Krausewitz@ukbonn.de)
- d.fostitsch@googlemail.com
- Richard.Weiten@ukbonn.de
- Niklas.Kluemper@ukbonn.de
- Johannes.stein@ukbonn.de
- Julian.Luetkens@ukbonn.de
- [glen.kristiansen@ukbonn.de](mailto:glen.kristiansen@ukbonn.de)
- [joerg.ellinger@ukbonn.de](mailto:joerg.ellinger@ukbonn.de)
- [mritter@ukbonn.de](mailto:mritter@ukbonn.de)

Supplementary Material

**Supplementary Table S1** Descriptive statistics of clinical measures

| Variable | All men (n=259) |
| --- | --- |
| Age (years) | 68.5 ± 11.8 |
| PSA (ng/ml) | 10.8 ± 5.2 |
| PSAD (ng/ml/cm³) | 0.27 ± 0.13 |
| Prostate volume (cm³) | 57.7 ± 34.0 |
| Abnormal DRE (%) | 43.0 |
| Abnormal US (%) | 29.4 |
| PI-RADS 3  PI-RADS 4  PI-RADS 5 | 42 (16.2)  137 (52.9)  80 (30.9) |
| Abnormal DRE  Normal DRE  Missing data | 107 (43.0)  142 (57.0)  10 |
| Suspicious US findings  Non-suspicious US  missing data | 70 (29.4)  168 (70.6)  21 |
| Target (418) location by PI-RADS sector map:  TZ  PZpl  PZpm  PZa  AS | 130 (31.1)  111 (26.6)  101 (24.2)  68 (16.3)  8 (1.9) |
| Target (418) distribution  Basis  Mid zone  Apex | 61 (14.6)  195 (46.7)  162 (38.8) |
| Time of intervention (min) | 11.3 ± 5.0 |
| Number of biopsy cores / patient | 13.7 ± 1.0 |
| CDR CB:  PCA (%)  csPCA (%)  nsPCA (%) | 71.8  65.6  6.2 |

**Supplementary Table S1** shows means and interquartile range or valid percentages of the collected patient data.

PSA, prostate specific antigen; PSAD, prostate specific antigen density; DRE, digital rectal examination; US, transrectal ultrasound; PI-RADS, The Prostate Imaging - Reporting and Data System Version 2 (PI-RADS™ v2.1); TZ, transitional zone of the prostate; PZpl, posterolateral zone of the peripheral prostate; PZpm, posteromedial zone of the peripheral prostate; PZa, anterior zone of the peripheral prostate; AS, anterior stroma of the prostate; CDR, cancer detection rate; PCA, prostate cancer; csPCA, clinically significant prostate cancer defined as Gleason ≥ 3+4; nsPCA, non-clinically significant caner defined as Gleason < 6

**Supplementary Table S2** 30 day’s complication rate after prostate biopsy

|  | Patients (%) | Clavien-Dindo Classification | Intervention |
| --- | --- | --- | --- |
| Post interventional gross hematuria | 1 (0.4%) | I | No further intervention required |
| Post interventional urinary tract infection | 2 (0.8%) | II | 1x oral antibiotic therapy  1x intravenous antibiotic therapy |
| Post interventional urinary retention | 3 (1.2%) | IIIa | Placement of urethral catheter |

**Supplementary Table S2** shows the retrospectively collected complication rate within 30 days after combined systematic (SB) and MRI-targeted (TB) prostate biopsy. Complications were graded according to Clavien-Dindo classification. Categorical data are presented as numbers %.

**Supplementary Table S3** Cancer detection rates according to biopsy method

| Variable | **TB (259)** | **SB (259)** | **CB (259)** |
| --- | --- | --- | --- |
| ISUP (Gleason)  No cancer  1 (6)  2 (7a)  3 (7b)  4 (8)  5 (9 & 10) | 104 (40.2)  17 (6.6)  67 (25.9)  35 (13.5)  11 (4.2)  25 (9.7) | 79 (30.5)  28 (10.8)  69 (26.6)  43 (16.6)  18 (6.9)  22 (8.5) | 73 (28.2)  16 (6.2)  72 (27.8)  51 (19.7)  20 (7.7)  27 (10.4) |
| CDR PCA (%) | 59.8 | 69.5 | 71.8 |
| CDR csPCA (%) | 53.2 | 58.7 | 65.6 |
| CDR nsPCA (%) | 6.6 | 10.8 | 6.2 |
| Tumor bearing biopsy cores | 238 (54.3) | 755 (24.3) | 993 (28.0) |
| Non-tumor tissue bearing biopsy cores | 200 (45.7) | 2353 (75.7) | 2553 (72.0) |
| Total biopsy cores | 438 (100.0) | 3108 (100.0) | 3546 (100.0) |

**Supplementary Table S3** shows cancer detection rates according to biopsy method: Magnetic resonance imaging targeted biopsy of the prostate (TB), 12-core systematic biopsy of the prostate (SB) and the combined approach of TB + SB (CB). Categorical data are presented as numbers %.

ISUP, International Society of Urological Pathology; CDR, cancer detection rate; PCA, prostate cancer; csPCA, clinically significant prostate cancer defined as Gleason ≥ 3+4; nsPCA, non-clinically significant caner defined as Gleason <6

**Supplementary Fig S1** Institutional standardized 12-core systematic biopsy


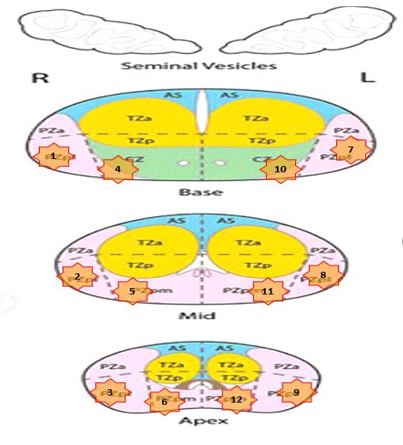


**Supplementary Fig. S1** illustrates the study scheme for institutional standardized 12-core systematic biopsy (SB) of the prostate used in all patients in the same setting as MRI-targeted biopsy was performed. A pre-defined, software-assisted template was used for SB. Due to standardization, cores 1/2/3/7/8/9 are representative for the outer peripheral posterolateral zone (PZpl) and the cores 4/5/6/10/11/12 are representative for the outer peripheral posteromedial zone (PZpm) and the central zone (CZ) defined by PIRADS v2.1 sector map [12].

**Supplementary Fig S2** Cancer detection of CB stratified by PCA surrogate markers


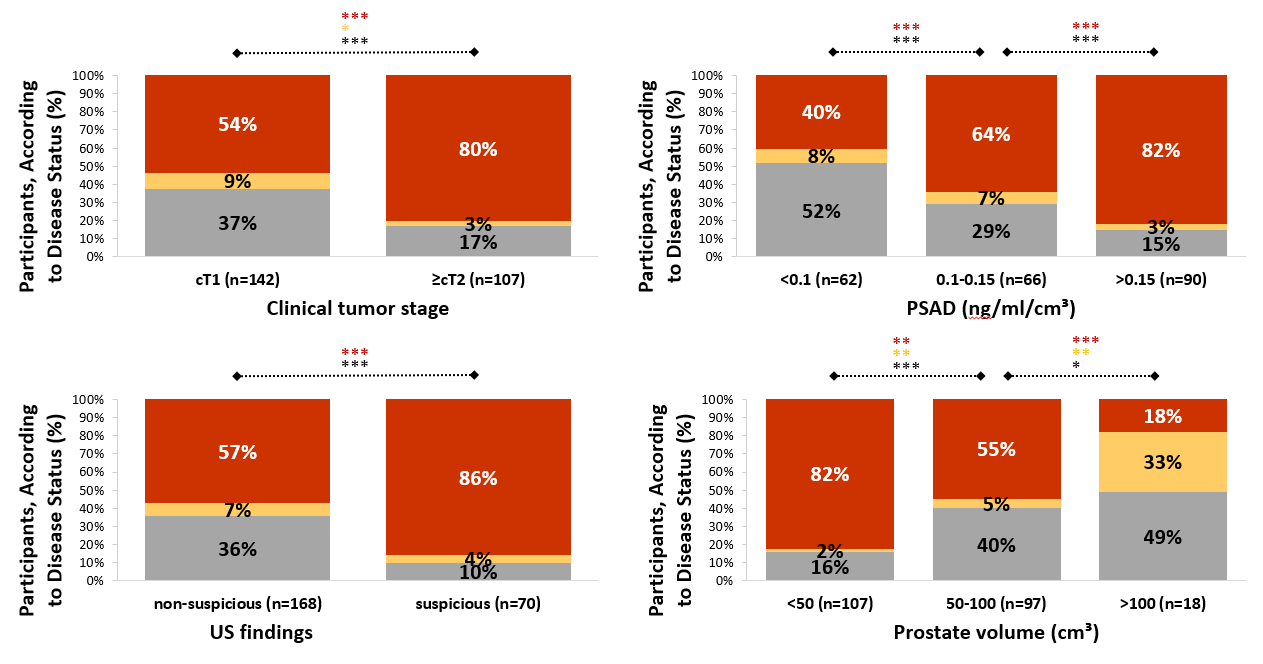


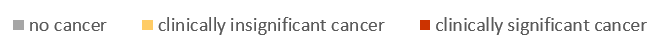


*** p < 0.001, ** p <0.01, * p < 0.05

**Supplementary Fig. S2** illustrates cancer detection rates by combined approach of targeted biopsy and systematic biopsy stratified by prostate volume, clinical tumor stage (cT1=non-suspicious digital rectal examination (DRE); cT2 abnormal DRE, Transrectal ultrasound (US) findings, and prostate specific antigen density (PSAD).

**Supplementary Fig. S3** Cancer detection rate per core taken by systematic biopsy


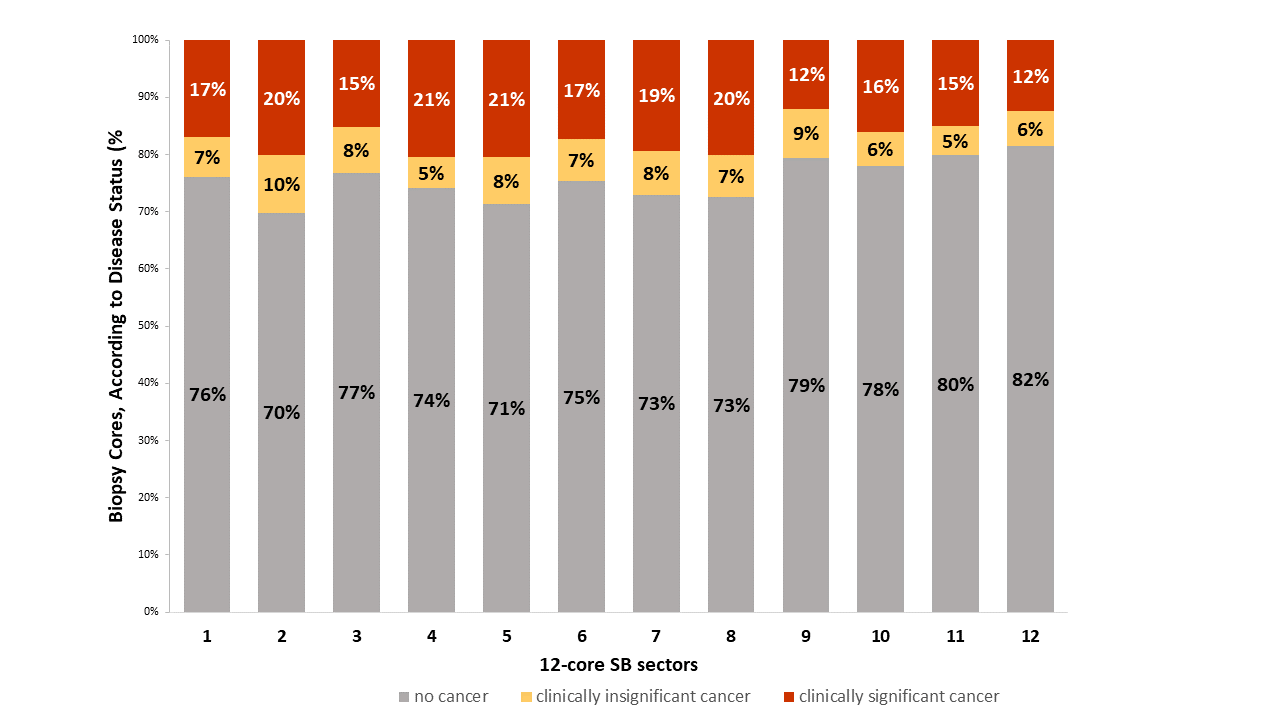


**Supplementary Fig. S3** shows cancer detection rates per biopsy core taken at standardized systematic biopsy (SB, core 1-12). Due to standardization, cores 1/2/3/7/8/9 are representative for the outer peripheral posterolateral zone (PZpl) and the cores 4/5/6/10/11/12 are representative for the outer peripheral posteromedial zone (PZpm) and the central zone (CZ) defined by PIRADS v2.1 sector map [12] as described in Supplementary Fig. 1. SB cores achieved CDR of PCA and csPCA of mean 24.3% ± 3.4 and 16.5% ± 4.0, respectively. Moreover, SB determined an equivocal amount of nsPCA in cores 1-12 (mean 7.5% ± 2.5)

**Supplementary Fig. S4** Cancer detection rate dependent on MRI index lesion distribution


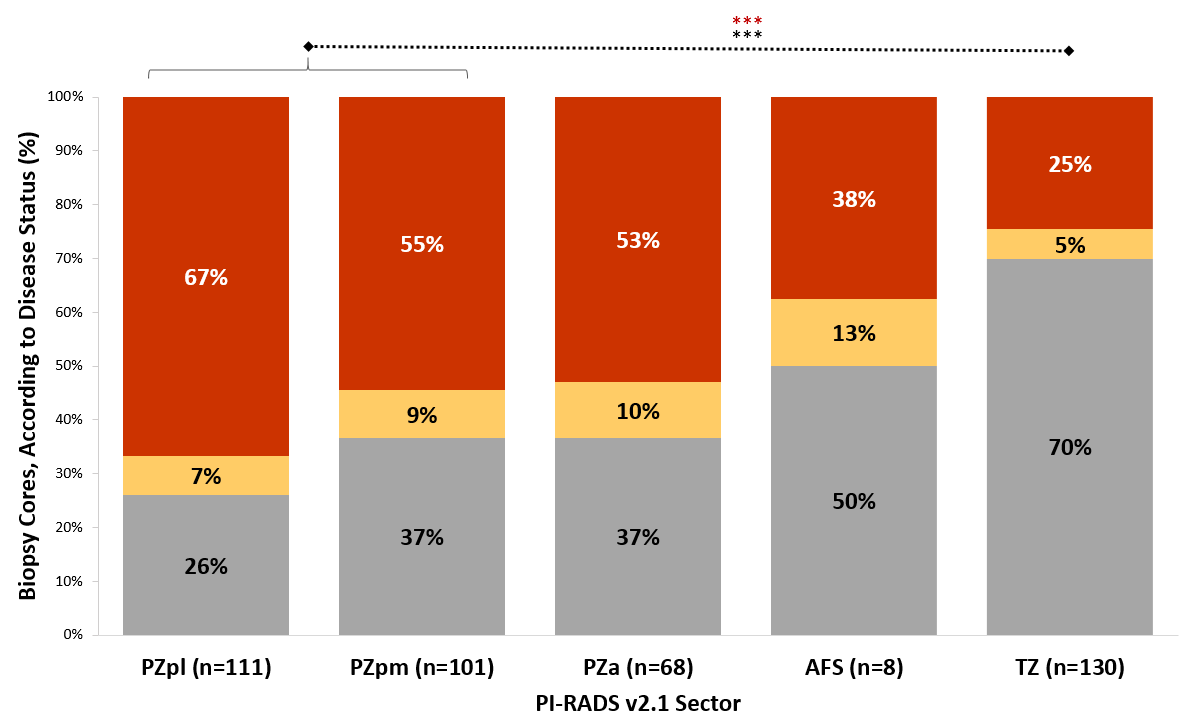


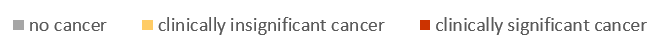


**Supplementary Fig. S4** illustrates cancer detection rates per biopsy core taken at different target locations defined defined by PIRADS v2.1 sector map [12].

PI-RADS, The Prostate Imaging - Reporting and Data System Version 2 (PI-RADS™ v2.1); TZ, transition zone of the prostate; PZpl, outer peripheral posterolateral zone of the peripheral prostate; PZpm, outer peripheral posteromedial zone of the prostate; PZa, anterior zone of the peripheral prostate; AFS, anterior fibromuscular stroma of the prostate
